# Exploring real-world acute associations between physical activity and bodily pain in middle-aged and older adults

**DOI:** 10.1097/PR9.0000000000001407

**Published:** 2026-03-03

**Authors:** Monica Teegardin, Anthony Kaleth, Priscilla Stone, Taylor Kelly, Easton Hewitt, Kelly Naugle

**Affiliations:** a Indiana University Indianapolis, Indianapolis, IN, USA; b Indiana University, Bloomington, IN, USA

**Keywords:** Moderate to vigorous physical activity, Musculoskeletal pain, Kinesiophobia, Sedentary behavior, Elderly

## Abstract

**Introduction:**

Acute real-world associations between moderate-to-vigorous physical activity (MVPA), sedentary behavior, and daily fluctuations in pain levels of older adults are not well-understood. **Objectives:** Using a real-world approach in middle-aged and older adults with musculoskeletal pain, the purpose of this observational study was to determine (1) within-person relationships between bodily pain intensity and PA levels in the hour prior and (2) whether the within-person relationships were moderated by typical pain levels and kinesiophobia.

**Methods:**

Participants (n = 46, age 55–85 years) completed questionnaires including the Graded Chronic Pain Scale (typical pain levels) and Tampa Scale of Kinesiophobia. Participants wore an accelerometer for 7 consecutive days to measure MVPA and sedentary time. During the same 7-day period, participants completed daily ecological momentary assessments (EMAs) of their bodily pain on a 0 to 10 scale using a smartphone app. Linear mixed models examined within-person associations between EMA pain intensity rating and the prior hour's PA levels and cross-level interactions.

**Results:**

Greater sedentary minutes in the hour before the EMAs predicted greater bodily pain, with characteristic pain moderating this relationship. A positive relationship was shown between sedentary minutes and pain intensity at higher characteristic pain levels. Kinesiophobia moderated the effect of hourly MVPA minutes on bodily pain. For those with relatively lower kinesiophobia, greater minutes in MVPA predicted lower pain; however, no relationship existed between MVPA and pain for those with higher kinesiophobia.

**Conclusion:**

Hourly MVPA acutely influenced subsequent bodily pain in the natural environment of middle-to-older aged adults with musculoskeletal pain.

## Abstract 2

Hourly physical activity levels acutely influenced subsequent bodily pain in the natural environment of middle-to-older aged adults with musculoskeletal pain.

## 1. Introduction

Epidemiological studies indicate a high prevalence of persistent pain in older adults, with estimates ranging from 60% to 75%.^6,32^ Most pain in older adults is musculoskeletal in nature and present at multiple body sites, with the most common location being the knees, low back, and hips.^3^ Regular physical activity (PA) is a well-accepted approach for the management of musculoskeletal pain and is often recommended as an adjunct to pharmacotherapy.^4,10,33^ An acute bout of exercise or moderate-to-vigorous PA (MVPA) can also influence the experience or perception of pain. Research in healthy, pain-free young adults indicates that a single bout of MVPA or isometric exercise induces a temporary period of hypoalgesia (<30 minutes).^20^ However, evidence suggests that older adults and some chronic pain populations may exhibit diminished or no hypoalgesia after acute MVPA compared with younger adults.^11,14,21,23,29^ Most research to date has examined the acute effects of MVPA on pain perception in a controlled laboratory environment. Little is known regarding how these effects may translate to the natural environment and whether MVPA performed throughout the day can have a “real world” beneficial influence on pain in those experiencing musculoskeletal pain. Furthermore, factors that moderate the impact of acute MVPA on pain also need to be identified. For example, kinesiophobia or the fear of movement or (re)injury related to pain has been shown to be associated with reduced levels of MVPA^12,19^ and predict discomfort during PA in older adults.^18^

Pain assessments that fully represent an individual's real-world pain experience are vital for pain management and research. To capture this everyday pain experience, ecological momentary assessments (EMAs) of pain have recently gained popularity.^9,28^ With EMA, pain intensity can be rated in real-time multiple times throughout the day to allow for the evaluation of the dynamic fluctuations of pain in an individual's natural environment.^31^ Ecological momentary assessment of pain ensures greater ecological validity by reducing measurement bias associated with retrospection and allowing investigations of within-person relationships between pain intensity and other contextual factors that may alleviate or exacerbate pain (eg, PA).^30^ Research looking at the real-time relationships between daily pain and MVPA in older adults have demonstrated positive daily associations between pain and activity and have shown the associations to be variable and dynamic.^2,7^ However, as most prior research of this nature has examined daily relationships between pain and MVPA, further research is needed to elucidate the short-term effects of MVPA and sedentary behavior on pain in daily life for older adults.

Using a real-world approach in middle-aged and older adults with musculoskeletal pain, the purpose of this study was to explore hourly within-person relationships between sedentary behavior and MVPA and subsequent bodily pain intensity in the natural environment. Specifically, we examined whether MVPA and sedentary levels in the hour before each momentary pain intensity rating were associated with the individual pain rating. The second purpose was to determine whether the within-person relationships were moderated by typical pain levels and fear of movement due to pain (ie, kinesiophobia).^32^ We hypothesized that sedentary behavior would be associated with higher pain intensity ratings, whereas greater MVPA would be associated with lower pain intensity ratings.

## 2. Methods

### 2.1. Procedures

All procedures for this observational research study were approved by the Indiana University Institutional Review Board, and all participants provided written informed consent. All study sessions took place in a private space in the Physical Activity and Pain Laboratory or a local community center. Participants completed one study session and a brief follow-up session. During the initial study session, participants gave informed consent, completed a healthy history assessment, and completed questionnaires including the Tampa Scale of Kinesiophobia, Graded Chronic Pain Scale, and Pain Body Map. Participants also were given an accelerometer to wear for 7 days, an accelerometer diary, and were asked to complete EMAs using a smartphone app for the same period. After 7 days, participants returned the accelerometer and diary, uninstalled the smartphone app, and received compensation for participation in the study.

### 2.2. Participants

Fifty-four community-dwelling adults (18 men, 36 women) aged 55 to 85 years enrolled in this study. The recruitment period was from November 2021 to May 2024. Participants were recruited from Indianapolis and surrounding areas by word of mouth, social media, flyers, local fitness centers, and email. All participants had access to an android phone or iPhone and reported experiencing musculoskeletal pain within the past month. Exclusion criteria included reporting any of the following: known cardiovascular disease; uncontrolled blood pressure; orthopedic, musculoskeletal, or neurological disorders that restricted normal daily activity; schizophrenia or other psychosis; diagnosed peripheral neuropathy; and chronic opioid use. No participants failed screening.

Because this study was exploratory, a power analysis was not conducted. However, when using multilevel models with maximum likelihood methods, guidelines suggest having a sample size of n ≥ 30 at level 2 (number of participants) of the model, with a level-1 sample size of n ≥ 30 (hourly PA data for each participant for this study).^16^ Another guideline suggested a level-2 sample size of approximately 50, with a level-1 sample size of n ≥ 20.^8,16^

### 2.3. Outcome measures

#### 2.3.1. Questionnaires

##### 2.3.1.1. Tampa Scale of Kinesiophobia-11

The Tampa Scale of Kinesiophobia (TSK) measures fear of movement or reinjury due to pain,^13^ consisting of 11 items rated on a 4-point Likert scale. Tampa Scale of Kinesiophobia scores range from 11 to 44, with higher scores indicating greater fear of movement. The TSK is a valid and reliable method for determining fear of movement in clinical and nonclinical populations.^5,34^

##### 2.3.1.2. Graded Chronic Pain Scale

The Graded Chronic Pain Scale (GCPS) was used to measure clinical pain the participant may have experienced in the past 6 months. Specifically, the GCPS is used to measure pain-related severity and disability across 7 items.^35^ Pain intensity is measured with the Characteristic Pain Intensity subscale (scores range from 0 to 100). Characteristic pain intensity is derived from 3 questions that assess *pain right now*, *worst pain*, and *average pain* on a 0 to 10 scale. The Disability subscale scores range from 0 to 100 and are derived from 4 questions assessing how much pain has interfered with various activities and work on a 0 to 10 scale. The Characteristic Pain Intensity subscale represented the typical pain level over the past 6 months and was used in the statistical analysis. The GCPS has been deemed a valid and reliable tool for assessing characteristic pain intensity, pain interference, and pain-related disability.^24^

##### 2.3.1.3. Pain body map

All participants completed a validated pain body map.^27^ Men and women completed separate maps, each with 2 side-by-side anterior and posterior representations of the body with a line dividing the right and left sides. The body map included 74 body regions. Participants shaded in the locations on the body map where they had experienced pain in the past 7 days. The total number of body regions represents the sum of all shaded regions.

#### 2.3.2. Objective assessment of physical activity

Participants wore an ActiGraph wGT3X-BT triaxial accelerometer (ActiGraph, Pensacola, FL) on their right hip for 7 consecutive days to measure real-time MVPA levels and sedentary behavior. The ActiGraph wGT3X-BT detects triaxial accelerations in the range of 0.05 to 2 G and provided step counts, body positions, and activity counts for a specified period. Data were captured in 60-second epochs, and a valid collection day was defined as ≥10 h/d of wear.^17^

Data download, reduction, cleaning, and analysis were conducted using the ActiLife 6 Data Analysis Software. The Sasaki et al.^26^ cut points based on vector magnitude were used to classify MVPA and sedentary behavior. For each valid day, participants' MVPA and sedentary time were calculated. We also extracted MVPA measures and sedentary time for each hour before each EMA assessment. For example, if the EMA assessment was captured at 10:15 am, we calculated the minutes in MVPA and sedentary time from 9:15 am to 10:15 am

Participants were provided an accelerometer diary to record the start and stop times for wearing the accelerometer each day, and the duration and reasons for periods the accelerometer was removed. They also recorded any pain medications taken during the data collection period, including the time taken. The accelerometer diary was completed during the same 7-day period the accelerometer was worn.

#### 2.3.3. Ecological momentary assessment of pain

Participants completed daily EMAs of bodily pain for one week using a smartphone app. The daily EMA was collected concurrently with the 7-day period during which participants wore the accelerometer and completed the diary.

##### 2.3.3.1. Technology to capture ecological momentary assessment

Ecological momentary assessment of pain was electronically delivered through Digibiomarkers (DigiBio, LLC, Carmel, IN), a HIPAA-compliant, cloud-based mobile and web application platform for researchers by MavenSphere, Inc. During the registration process, participants were instructed to turn notifications “ON” to receive prompts from the DigiBio app throughout the day. On completion of the study, participants were asked to uninstall the app.

##### 2.3.3.2. Schedule of assessments

Pain was assessed in 6 predetermined hourly segments throughout the day. Notifications were administered on the hour and participants had 1 hour to complete the assessment (eg, EMA prompted at 10:00 am; participant had until 11:00 am to complete the assessment). To ensure a representative sample of each respondents' daily activities, the segments were distributed in the morning, afternoon, and evening (10:00 am, 12:00 pm, 2:00 pm, 4:00 pm, 6:00 pm, 8:00 pm).

##### 2.3.3.3. Pain intensity item

The pain intensity item used a momentary approach by asking: “Please rate your bodily pain by selecting the one number that tells how much pain you have right now,” with 0 indicating “No pain” and 10 indicating “Pain as bad as you can imagine.” This question was adapted from the Brief Pain Inventory question assessing pain right now (“Please rate your pain by marking the box beside the number that tells how much pain you have right now,” with 0 indicating “No pain” and 10 indicating “Pain as bad as you can imagine”). The actual time of the pain rating was recorded.

### 2.4. Data analysis

Data were analyzed using IBM SPSS Statistics version 29. Participant data were first screened to identify participants who reported no-to-little pain on the EMAs and those who did not have sufficient data for data analysis. Specifically, participants were excluded from data analysis if: (1) they reported pain (>0) on less than 10% of their EMAs (n = 5) or (2) they did not have at least 4 days of 3 EMAs completed that included matching hourly PA data (n = 3). Therefore, the final analysis included data on 46 participants. Most of the 8 excluded participants were male (6/8), White (6/8), and significantly older (70.8 ± 7.6 vs 63.0 ± 5.9, *P* = 0.022). The Characteristic Pain Intensity score of the GCPS did not significantly differ between those included (36.7 ± 17.5) vs excluded (27.1 ± 18.1, *P* = 0.157).

Linear mixed model was used as the primary analysis. The model allowed the random intercept to vary and used an AR-1 covariance structure. Day and pain rating were specified as repeating variables. The dependent variable was the EMA pain intensity rating. The level-1 variables included the hour-before the EMA minutes of MVPA (hourly MVPA) and sedentary time (hourly sedentary time). At level-1, we examined within person associations between EMA pain intensity rating and the prior hour's PA derived from the accelerometers. The level-1 variables were person-mean centered. Level-2 variables included sex, age, characteristic pain intensity (GCPS—intensity subscale score), TSK scores, and average daily MVPA. Level-2 variables were converted into Z scores. In addition, we tested the cross-level interactions to examine potential moderating roles of characteristic pain intensity and TSK. Specifically, we tested the cross-level interactions between (1) each of the level-1 predictors (MVPA hourly, sedentary hourly) with characteristic pain intensity, and (2) MVPA hourly with TSK score. Simple slope analyses were used to follow-up significant interactions. The linear mixed models used maximum likelihood estimation.

Participants self-reported the pain medications taken during the data collection period on the accelerometer diary. According to these data, 6 participants took prescription pain medications on a regular schedule. We also examined the self-report diary data to determine which EMAs could have been influenced by pain medication based on the timing of the meds and EMA. Not including the regular prescription medication, 53 EMAs (across the 46 participants) that included corresponding MVPA and sedentary data were recorded after a participant took a pain medication (eg, any EMA recorded on a day after taking Tylenol or Aleve). This represented only 3.5% of the EMA data. To ensure that the pain medications did not influence the data, we conducted the linear mixed models described above with (1) the 53 EMAs removed from the data set and (2) the 53 EMAs and 6 participants taking prescription pain medications removed from the data set. The significant variables (as described in Results) did not change and coefficients were minimally altered with these data removed; thus, we included the 53 EMAs potentially affected by pain medications and the 6 participants taking prescription pain medications in the final analyses.

## 3. Results

Descriptive data for all participants and the primary outcome measures are summarized in Tables 1 and 2, respectively. In a summary of the Pain Map results, the most common pain sites included lower back (58.7%), knee (56.5%), hip (32.6%), hand (32.6%), and shoulder (37.0%). All but one participant reported pain in more than one body region. Participants completed an average of 33 ± 6.0 EMAs with corresponding MVPA and sedentary behavior data across the 7 days. Seventy-two percent of participants reported their highest EMA pain rating as ≥3, while 54% reported their highest EMA pain rating ≥4. Across all 46 participants, each day had an average of 215 ± 8.8 EMAs with corresponding MVPA and sedentary behavior data. Each EMA time point, across all days and participants, had an average of 251 ± 11.7 EMAs with corresponding MVPA and sedentary behavior data.

**Table 1:** Participant characteristics (N = 46).

Variable	Mean (SD) or %
Age, y	63.0 (5.9)
Sex, % female	73.9%
BMI, kg/m^2^	29.4 (5.8)
Education completed, %	
Some high school	2.2%
High school degree	13%
Two-year college degree	15.2%
Four-year college degree	15.2%
Master's or above	54.3%
Race, %	
African American	23.9%
Caucasian	69.6%
Hispanic	4.3%
Other	2.2%
Taking prescription	
Medications for pain, %	13%
No. of bodily pain sites	7.8 (5.4)

**Table 2:** Descriptive statistics N = 46.

Variable	Mean (SD)	Range
GCPS—characteristic pain intensity score	36.7 (17.5)	0–83.3
GCPS—pain disability score	23.5 (26.6)	0–96.7
Average TSK score	19.2 (5.6)	11–32
Average EMA pain rating	1.4 (1.6)	0–8
Average sedentary minutes per day	520.4 (93.2)	325.3–691.4
Average % wear time in sed min per day	59.5 (9.1)	36.6–80.6
Average MVPA per day	37.0 (23.9)	3.1–102.7
Average hourly sedentary minutes	36.0 (16.0)	0–60
Average hourly MVPA minutes	2.3 (5.6)	0–58

EMA, ecological momentary assessment; GCPS, Graded Chronic Pain Scale; MVPA, moderate to vigorous physical activity; sed, sedentary; TSK, Tampa scale of kinesiophobia.

### 3.1. Main effects of linear mixed model

Table 3 summarizes the within-person associations between the EMA pain intensity rating and the prior hour's MVPA performed and sedentary time as derived from accelerometry. Hourly sedentary minutes and characteristic pain intensity predicted pain intensity ratings on the EMA. Hourly MVPA minutes approached significance. Greater sedentary minutes the hour before the EMA predicted greater intensity of pain. As expected, higher levels of characteristic pain intensity predicted greater EMA pain intensity ratings. The main effects of sex, age, average daily MVPA, and TSK were not significant.

**Table 3:** Main and interactive effects of the prior hour's physical activity on bodily pain intensity rating.

Predictor	Parameter estimate (95% CI)	*P*
Level 1		
Hourly MVPA	−0.009 (−0.019, 0.000)	0.058
Hourly sedentary time	0.007 (0.003, 0.011)	<0.001*
Level 2		
Sex	0.379 (−0.227, 0.986)	0.214
Age	0.225 (−0.033, 0.483)	0.086
Characteristic pain intensity	0.846 (0.562, 1.130)	<0.001*
TSK score	−0.028 (−0.304, 2.47)	0.836
Average daily MVPA	0.163 (−0.106, 0.432)	0.228
Interactions		
Hourly MVPA × characteristic pain level	−0.002 (−0.012, 0.008)	0.678
Hourly sedentary time × characteristic pain level	0.010 (0.006, 0.013)	<0.001*
Hourly MVPA × TSK score	0.017 (0.005, 0.028)	0.005*

* Significant at *P* < 0.05.

MVPA, moderate to vigorous physical activity; TSK, Tampa scale of kinesiophobia.

### 3.2. Moderating effects of characteristic pain intensity

**Figure 1.**
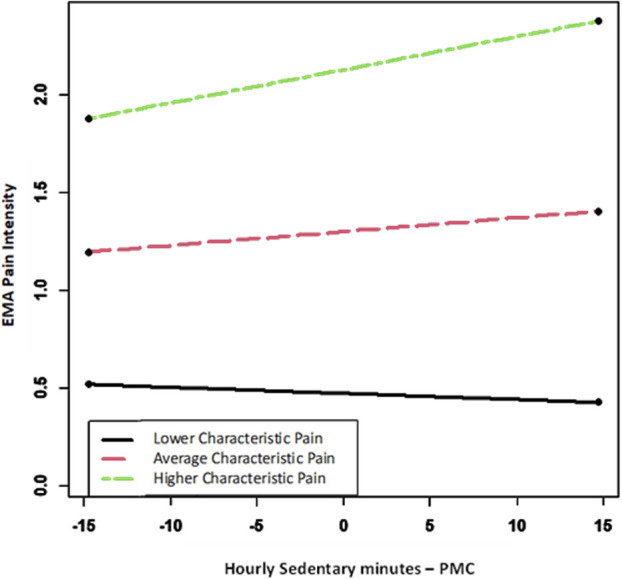
Interaction between characteristic pain level and hourly sedentary minutes on EMA pain intensity rating. EMA, ecological momentary assessment; PMC, person-mean centered.

The cross-level interaction of characteristic pain intensity and sedentary minutes was significant. As shown in Figure 1, characteristic pain intensity moderated the effect of hourly sedentary minutes on pain intensity ratings. Specifically, at the mean of characteristic pain intensity (β = 0.007 [0.0019]; *P* = 0.0002) and higher characteristic pain intensity (1 SD above the mean) (β = 0.017 [0.0026]; *P* < 0.0001) within the sample, a positive relationship existed between sedentary minutes and EMA pain intensity. Thus, for those with relatively higher characteristic pain intensity in the sample, greater minutes in sedentary time predicted greater EMA pain. At lower characteristic pain intensity (1 SD below mean), a significant relationship between sedentary time and EMA pain intensity did not exist (β = −0.003 [0.0026]; *P* = 0.24). The interaction between hourly MVPA and characteristic pain intensity was not significant.

### 3.3. Moderating effects of Tampa Scale of Kinesiophobia

**Figure 2.**
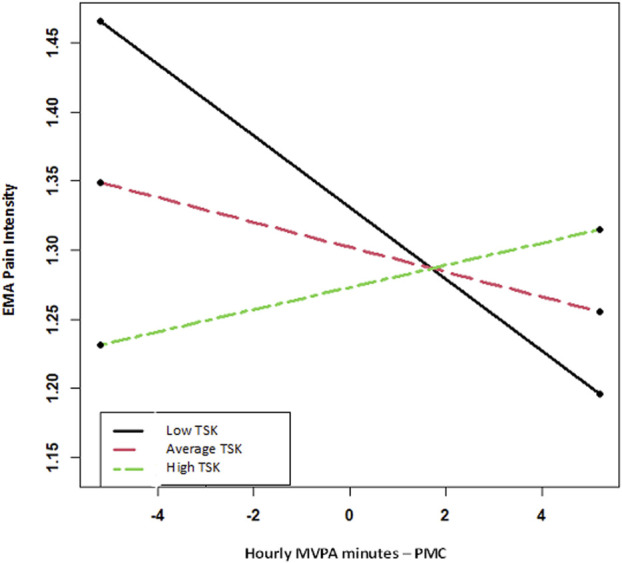
Interaction between kinesiophobia and hourly MVPA minutes on EMA pain intensity rating. EMA, ecological momentary assessment; MVPA, moderate to vigorous physical activity; PMC, person-mean centered; TSK, Tampa Scale of Kinesiophobia.

The cross-level interaction of TSK and hourly MVPA minutes was significant. As shown in Figure 2, the TSK score moderated the effect of hourly MVPA minutes on pain intensity ratings. Specifically, at relatively lower levels of TSK (1 SD below the mean), a negative relationship existed between MVPA minutes and pain intensity (β = −0.026 [0.0071]; *P* = 0.0002). Thus, for those with relatively lower TSK in the sample, greater minutes in MVPA predicted subsequent lower pain. At relatively higher levels of TSK (1 SD above mean: β = 0.008 [0.0082]; *P* = 0.33) and at the mean (β = −0.009 [0.0049]; *P* = 0.07), MVPA minutes did not significantly predict pain intensity.

## 4. Discussion

Acute real-world associations between MVPA, sedentary behavior, and daily fluctuations in pain levels of adults are not well-understood. This study used a novel real-word approach to explore how pain intensity relates to MVPA patterns and sedentary behavior in the hour prior. We also explored whether usual pain levels and kinesiophobia moderated this relationship. Several key findings materialized from the results. First, hourly sedentary minutes and characteristic pain predicted pain intensity ratings on EMA, but hourly MVPA minutes did not reach significance. Second, greater minutes in sedentary time predicted greater pain for participants who reported relatively higher characteristic pain. However, a significant relationship did not exist between sedentary time and EMA pain intensity for participants who reported lower characteristic pain levels. Third, kinesiophobia moderated the effect of hourly MVPA minutes on EMA pain intensity ratings. Specifically, a negative relationship existed between MVPA minutes and EMA pain intensity at relatively low levels of kinesiophobia.

This study demonstrated that a longer duration of sedentary behavior in the hour before the EMA predicted greater levels of pain. Although this result aligns with our hypothesis, it stands in contrast to previous research. Park et al. determined that greater daily sedentary behavior was associated with a reduced level of pain in the evening.^25^ It is possible the reduced pain was a result of moving less throughout the day. Age may have also played a role in the discrepancy with the findings as the average age of participants in this study was much younger. Moreover, characteristic pain intensity moderated the effects of hourly sedentary minutes on EMA pain intensity. Greater sedentary minutes predicted greater EMA pain for participants with higher characteristic pain levels. However, no significant relationship existed between sedentary minutes and EMA pain intensity for those at lower characteristic pain levels. Further research is warranted to determine the short-term effects of sedentary behavior on pain levels. Interestingly, prior work has linked sedentary behavior to the capacity of the central nervous system to endogenously inhibit pain (a risk factor for more persistent and intense pain) in older adults.^22^ However, it is unclear whether this relationship is driven by short-term changes in pain inhibitory capacity after acute bouts of sedentary behavior (eg, hours of sitting) or long-term adaptations in the central nervous system secondary to habitually sedentary behavior. Future research should investigate whether bouts of sitting cause short-term declines in endogenous pain inhibition that translate to increased bodily pain.

This study revealed a significant cross-level interaction of TSK and hourly MVPA minutes. At relatively lower levels of kinesiophobia, greater MVPA was associated with subsequently lower pain intensity ratings. Because most of the MVPA minutes consisted of moderate rather than vigorous PA, this relationship was likely driven by moderate intensity PA. It should also be noted that pain intensity was not measured at the beginning of the hour; therefore, it cannot be concluded that MVPA predicted a reduction in pain, but only that an association existed with lower levels of pain. By contrast, at higher levels of kinesiophobia, hourly MVPA minutes were not associated with lower pain ratings. The relationship even trended in the opposite direction at the highest levels of kinesiophobia, but did not reach significance. Notably, the TSK scores of our sample were relatively low (average∼19, range 11–32), but in line with scores from another large study of older adults with heterogenous chronic pain.^15^ Similar to our study, Miller et al.^18^ determined kinesiophobia predicted sensitivity to PA during a 6-minute walking task and concluded that higher levels of kinesiophobia predicted a greater increase in whole-body discomfort during walking. Furthermore, Brellenthin et al.^1^ revealed during a laboratory study that psychosocial variables (ie, situational catastrophizing and mood) predicted muscle pain during exercise and EIH, with worse mood and catastrophizing relating to greater pain and worse EIH outcomes. It can be argued that kinesiophobia may interfere with the potential hypoalgesic effects of MVPA, but further research is needed. Collectively, these results could explain why kinesiophobia is associated with lower levels of MVPA in older adults.^19^

Unlike a previous study, this study did not find any effects of sex. Ho et al.^7^ conducted an observational, real-world study and determined that greater daily pain aggregated across 3 daily pain ratings was associated with increased daily MVPA in women, but not men. While this contradicts the current findings of this study, a higher sample size may have been needed to determine any moderating effects of sex. This study's findings are also in contrast to Park et al. which demonstrated that MVPA throughout the day predicted the intensity of bodily pain rated in the evening of older adults, with greater daily MVPA associated with greater pain.^25^ Notably, the Park et al. and Ho et al. studies did not consider psychological moderators of the PA–pain relationship and examined daily associations while our study focused on hourly associations between MVPA and pain.^7,25^ Laboratory studies on EIH suggest that the pain-reducing effects of MVPA typically last no longer than 30 minutes.^20^ Therefore, examining hourly associations may provide a clearer understanding of the relationship between physical activity, pain, and sedentary behavior.

A few limitations of this study should be noted. First, our participant sample had low to moderate levels of pain, and the results could be different in adults with higher pain levels. Second, MVPA levels for our participant sample were relatively high. Third, a pain rating at the beginning of the hour was not collected, making it possible that individuals who were in greater pain moved less. Fourth, our participant sample was predominately well-educated women which affects the generalizability to broader populations of middle-aged and older adults with lower socioeconomic status and individuals who are male. Future studies should include a larger sample size and more biological men. Fifth, while accelerometers can provide an objective measure of PA, limitations do exist. Hip-worn accelerometers primarily measure locomotor activity and are unable to capture upper-body activity. Accelerometers are also unable to distinguish between an individual carrying any weight and they are unable to distinguish between changes in body position (ie, sitting vs standing still). Sixth, while EMAs can provide real-time data, there is the potential for measurement errors such as delays in response and self-report biases. Finally, our participants reported musculoskeletal pain in many different bodily regions; therefore, the results cannot be specified for one type of pain. However, this is also a strength of the study, as our sample likely represents the typical clinical pain experience for older adults. Evidence suggests that more than half of older adults experience musculoskeletal pain in several body sites at once, with the most common sites being the knees, low back, and hips.^3^ Participants in our study reported experiencing pain in an average of 7 different body locations on the pain map, with the most common locations being the knee, low back, hip, and shoulder. Despite this variation in pain experiences within our sample, we still showed a within-person relationship between hourly MVPA and bodily pain. Possibly, stronger real-time relationships between MVPA, sedentary behavior, and pain may exist when measured in a specific pain population.

In summary, this study provides novel evidence for the acute relationships between MVPA, sedentary behavior, and subsequent bodily pain in the natural environment of middle-aged and older adults with musculoskeletal pain. Indeed, this study is the first to examine real-time hourly relationships between bodily pain and MVPA. Our findings highlight that sedentary behavior and PA are distinct concepts and can have differing relationships with pain. Based on the current results, future research should investigate whether short periods of sedentary behavior elicit a hyperalgesic effect in middle-aged and older adults. In addition, while our findings indicate that greater hourly MVPA is linked to lower subsequent bodily pain in those with a lower fear of movement, previous research has shown positive daily relationships between MVPA and pain in older adults.^7,25^ Therefore, future research should explore whether an optimal amount or pattern of MVPA exists for middle-aged and older adults that maximizes its short-term pain-relieving benefits while minimizing the risk of increased pain later in the day. Finally, our results suggest that fear of movement even at relatively low to moderate levels may negatively affect the short-term relationship between MVPA and pain, making kinesiophobia a potential target for pain management interventions.

## Disclosures

The authors have no conflicts of interest to declare.
